# Draft Genome Sequence of the d-Xylose-Fermenting Yeast *Spathaspora arborariae* UFMG-HM19.1A^T^

**DOI:** 10.1128/genomeA.01163-13

**Published:** 2014-01-16

**Authors:** Francisco P. Lobo, Davi L. Gonçalves, Sergio L. Alves, Alexandra L. Gerber, Ana Tereza R. de Vasconcelos, Luiz C. Basso, Glória R. Franco, Marco A. Soares, Raquel M. Cadete, Carlos A. Rosa, Boris U. Stambuk

**Affiliations:** Laboratório Multiusuário de Bioinformática, Embrapa Informática Agropecuária, Campinas, São Paulo, Brazila; Departamento de Bioquímica, Universidade Federal de Santa Catarina, Florianópolis, Santa Catarina, Brazilb; Laboratório de Bioinformática, Unidade de Genômica Computacional Darcy Fontoura de Almeida, Laboratório Nacional de Computação Científica, Petrópolis, Rio de Janeiro, Brazilc; Departamento de Ciências Biológicas, Escola Superior de Agricultura Luiz de Queiroz, Universidade de São Paulo, Piracicaba, São Paulo, Brazild; Departamento de Bioquimica, Universidade Federal de Minas Gerais, Belo Horizonte, Minas Gerais, Brazile; Departamento de Microbiologia, Universidade Federal de Minas Gerais, Belo Horizonte, Minas Gerais, Brazilf

## Abstract

The draft genome sequence of the yeast *Spathaspora arborariae* UFMG-HM19.1A^T^ (CBS 11463 = NRRL Y-48658) is presented here. The sequenced genome size is 12.7 Mb, consisting of 41 scaffolds containing a total of 5,625 predicted open reading frames, including many genes encoding enzymes and transporters involved in d-xylose fermentation.

## GENOME ANNOUNCEMENT

Yeasts of the genus *Spathaspora* have recently been discovered to be associated with rotting wood substrates and insects that occupy this ecological niche, and they produce typical elongated ascospores with curved ends (1–4). These yeasts are biotechnologically important because they can ferment d-xylose and other sugars present in plant biomass hydrolysates (2–6). The yeast *Spathaspora arborariae* was first isolated from rotting wood samples collected in the Atlantic rainforest and the Cerrado ecosystem in Brazil, and it not only ferments d-xylose efficiently, but it can also be used for bioethanol production from lignocellulosic hydrolysates (2, 7). Since d-xylose-fermenting yeasts are an important source of genomic information for biotechnological traits, including genes, enzymes, and/or sugar transporters to engineer industrial strains for the efficient production of bioethanol from renewable biomass (8), the draft genome of *S. arborariae* strain UFMG-HM19.1A^T^ was determined and automatically annotated.

Genome sequencing was performed by a whole-genome shotgun strategy using Roche 454 paired-end (insert size of 3 kb) technology. The raw sequence data comprise 915,700 reads with 657,682 mate-pairs totaling 291,670,584 nucleotides. The reads were assembled using Newbler (9) with default parameters into 439 contigs and 41 scaffolds, with a total length of 12,708,019 bp. The assembled genome has an N_50_ of ~679 kb (6 scaffolds) and an N_90_ of ~202 kb (18 scaffolds), with an average G+C content of 31.7%, which is comparable to those of other yeast genomes (10). We performed genome annotation with MAKER2 (11) and found 5,625 putative open reading frames (ORFs) >100 nucleotides (nt), in agreement with the genome content of other yeast species of the CTG clade (this taxon translates the CTG codon as serine instead of leucine [12]). Automatic gene annotation using the BLAST software (13) revealed 5,402 ORFs similar to sequences in the nonredundant protein database from the National Center for Biotechnology Information (e-value cutoff of 10^-10^). We identified 185 tRNA genes scattered across the scaffolds and located the 28S and 18S rRNA genes at scaffold 9 using tRNAscan-SE 1.3 (14) and RNAmmer (15) softwares, respectively. The *S. arborariae* mitochondrial genome was assembled into a 22,709-bp fragment (scaffold 29). Pulsed-field gel electrophoresis (16) revealed seven chromosomal bands, with one probably migrating as a doublet. Thus, the 40 scaffolds with approximately 12.7 Mb are likely to represent most of the genomic content found on the eight chromosomes present in this yeast.

The draft genome of *S. arborariae* revealed several interesting biotechnological genes for bioethanol production, including genes coding for sugar transporters and d-xylose reductase, xylitol dehydrogenase, and xylulokinase, responsible for the conversion of d-xylose to d-xylulose-5P, which is subsequently channeled into the pentose-phosphate pathway for ethanol production. The genes for d-xylose reductase from *Spathaspora* yeasts (*Spathaspora passalidarum* and *Spathaspora arborariae*) are particularly interesting, as these enzymes accept both NADH and NADPH as cofactors, allowing anaerobic d-xylose fermentation (5, 17).

### Nucleotide sequence accession numbers.

This whole-genome shotgun project has been deposited at DDBJ/EMBL/GenBank under the accession no. AYLH00000000. The version described in this paper is version AYLH01000000.
